# Supplementary material for: Mathematical Modelling of Cervical Precancerous Lesion Grade Risk Scores: Linear Regression Analysis of Cellular Protein Biomarkers and Human Papillomavirus E6/E7 RNA Staining Patterns
Source: Diagnostics (Basel). 2023 Mar 13;13(6):1084. doi: 10.3390/diagnostics13061084 (PMC10047622; doi:10.3390/diagnostics13061084)
Supplement: Supplementary file 1 [file diagnostics-13-01084-s001.zip › diagnostics-2148361-supplementary.pdf]

Supplement Table S1. Experimental results of substituting values in the development of mathematical models.

No.	Age	Age group	Grade	Ki−67 AS	Ki−67 P/N	p16 AS	p16 P/N	CTTN AS	CTTN P/N	mRNA *E6E7*
1	49	3	normal	0	1	0	1	2	1	1
2	43	3	normal	0	1	0	1	0	1	1
3	57	4	normal	0	1	0	1	3	1	1
4	27	1	normal	0	1	0	1	3	1	1
5	49	3	normal	0	1	0	1	3	1	2
6	42	3	normal	0	1	0	1	3	1	2
7	44	3	normal	0	1	0	1	4	2	1
8	54	4	normal	0	1	0	1	5	2	2
9	43	3	normal	0	1	0	1	7	2	2
10	54	4	normal	0	1	0	1	5	2	2
11	51	4	normal	3	1	0	1	0	1	2
12	36	2	normal	3	1	0	1	3	1	2
13	36	2	normal	4	2	0	1	3	1	2
14	43	3	normal	4	2	0	1	0	1	2
15	57	4	normal	5	2	0	1	3	1	2
16	51	4	normal	4	2	0	1	6	2	2
17	38	2	normal	4	2	0	1	6	2	2
18	42	3	normal	4	2	0	1	6	2	2
19	35	2	normal	4	2	0	1	4	2	2
20	46	3	normal	5	2	0	1	5	2	2
21	48	3	LSIL	0	1	0	1	0	1	2
22	47	3	LSIL	0	1	0	1	0	1	2
23	20	1	LSIL	0	1	0	1	2	1	2
24	35	2	LSIL	0	1	2	1	6	2	1
25	49	3	LSIL	0	1	0	1	5	2	2
26	50	4	LSIL	0	1	0	1	4	2	2
27	61	5	LSIL	0	1	0	1	6	2	2
28	21	1	LSIL	0	1	0	1	7	2	2
29	48	3	LSIL	0	1	0	1	7	2	2

30	34	2	LSIL	0	1	0	1	4	2	2
31	36	2	LSIL	0	1	5	1	6	2	1
32	32	2	LSIL	0	1	2	1	6	2	2
33	54	4	LSIL	0	1	2	1	7	2	2
34	51	4	LSIL	0	1	2	1	6	2	2
35	34	2	LSIL	5	2	0	1	6	2	1
36	57	4	LSIL	0	1	5	1	6	2	2
37	84	5	LSIL	0	1	5	1	6	2	2
38	29	1	LSIL	4	2	0	1	4	2	2
39	23	1	LSIL	4	2	0	1	6	2	2
40	54	4	LSIL	5	2	0	1	6	2	2
41	24	1	LSIL	5	2	5	1	7	2	1
42	43	3	LSIL	4	2	4	1	4	2	2
43	39	2	LSIL	4	2	5	2	5	2	2
44	47	3	LSIL	5	2	5	1	6	2	2
45	51	4	LSIL	6	2	4	2	4	2	2
46	24	1	LSIL	6	2	4	2	6	2	2
47	35	2	HSIL	0	1	0	1	5	1	2
48	47	3	HSIL	0	1	5	1	4	2	2
49	51	4	HSIL	0	1	5	2	7	2	2
50	68	5	HSIL	6	2	7	2	2	1	2
51	55	4	HSIL	5	2	5	2	5	2	2
52	49	3	HSIL	5	2	5	2	5	2	2
53	51	4	HSIL	7	2	6	2	2	1	2
54	43	3	HSIL	5	2	6	2	7	2	2
55	61	5	HSIL	6	2	5	2	6	2	2
56	38	2	HSIL	6	2	5	2	6	2	2
57	47	3	HSIL	6	2	6	2	7	2	2
58	51	4	HSIL	6	2	6	2	7	2	2
59	32	2	HSIL	6	2	7	2	6	2	2
60	51	4	HSIL	6	2	7	2	7	2	2
61	39	2	HSIL	7	2	7	2	5	2	2
62	54	4	HSIL	7	2	7	2	7	2	2
63	34	2	HSIL	7	2	7	2	7	2	2

64	52	4	HSIL	7	2	7	2	7	2	2
65	33	2	HSIL	7	2	7	2	7	2	2
66	47	3	HSIL	7	2	7	2	7	2	2
67	47	3	HSIL	7	2	7	2	7	2	2
68	47	3	SCC	0	1	7	2	7	2	2
69	82	5	SCC	0	1	7	2	7	2	2
70	60	4	SCC	7	2	6	2	7	2	2
71	51	4	SCC	5	2	7	2	7	2	2
72	58	4	SCC	7	2	7	2	7	2	2
73	62	5	SCC	7	2	7	2	7	2	2
74	45	3	SCC	7	2	7	2	7	2	2
75	55	4	SCC	7	2	7	2	7	2	2

P/N = positive or negative, AS = Allred scored, CTTN = cortactin

Supplement Table S2. The expected prediction value from development of mathematical models.

No.	Age	Age group	Grade	model 1	model 2	model 3	model 4	model 5
1	60	4	normal	1.15	0.384	0.211	1.065	0.1
2	49	3	normal	1.15	0.384	0.211	1.065	0.253
3	43	3	normal	1.15	0.384	0.211	1.065	0.253
4	51	4	normal	1.15	0.384	0.211	1.065	0.1
5	48	3	normal	1.15	0.384	0.211	1.065	0.253
6	45	3	normal	1.15	0.384	0.211	1.065	0.253
7	48	3	normal	1.15	0.384	0.211	1.065	0.253
8	43	3	normal	1.15	0.384	0.211	1.065	0.253
9	48	3	normal	1.15	0.384	0.211	1.065	0.253
10	40	2	normal	1.15	0.384	0.211	1.065	0.406
11	55	4	normal	1.15	0.384	0.211	1.065	0.1
12	24	1	normal	1.15	0.384	0.211	1.065	0.559
13	50	4	normal	1.15	0.384	0.211	1.065	0.1
14	42	3	normal	1.15	0.384	0.211	1.065	0.253
15	33	2	normal	1.15	0.384	0.211	1.065	0.406
16	49	3	normal	1.15	0.384	0.729	0.785	0.77
17	43	3	normal	1.15	0.384	0.729	0.785	0.77
18	57	4	normal	1.15	0.384	0.729	0.785	0.617
19	27	1	normal	1.15	0.384	0.729	0.785	1.076
20	38	2	normal	1.15	0.954	0.768	1.595	0.945
21	42	3	normal	1.15	0.954	0.768	1.595	0.792
22	61	5	normal	1.15	0.954	0.768	1.595	0.486
23	50	4	normal	1.15	0.954	0.768	1.595	0.639
24	63	5	normal	1.15	0.954	0.768	1.595	0.486
25	50	4	normal	1.15	0.954	0.768	1.595	0.639
26	52	4	normal	1.15	0.954	0.768	1.595	0.639
27	47	3	normal	1.15	0.954	0.768	1.595	0.792
28	41	3	normal	2.226	1.452	1.191	2.613	1.054
29	33	2	normal	2.226	1.452	1.191	2.613	1.207

30	42	3	normal	2.226	1.452	1.191	2.613	1.054
31	50	4	normal	2.226	1.452	1.191	2.613	0.901
32	49	3	normal	1.15	0.384	1.247	0.505	1.287
33	42	3	normal	1.15	0.384	1.247	0.505	1.287
34	44	3	normal	1.15	0.954	1.286	1.315	1.309
35	44	3	normal	2.495	1.719	1.436	3	1.441
36	46	3	normal	2.226	2.022	1.748	3.143	1.593
37	48	3	normal	2.226	2.022	1.748	3.143	1.593
38	54	4	normal	1.15	0.954	1.804	1.035	1.673
39	43	3	normal	1.15	0.954	1.804	1.035	1.826
40	54	4	normal	1.15	0.954	1.804	1.035	1.673
41	51	4	normal	1.957	1.185	1.982	1.666	2.295
42	36	2	normal	1.957	1.185	1.982	1.666	2.601
43	55	4	normal	2.495	2.289	1.993	3.53	1.827
44	63	5	normal	2.495	2.289	1.993	3.53	1.674
45	49	3	normal	2.495	2.289	1.993	3.53	1.98
46	46	3	normal	2.495	2.289	1.993	3.53	1.98
47	36	2	normal	2.226	1.452	2.227	2.053	2.241
48	43	3	normal	2.226	1.452	2.227	2.053	2.088
49	52	4	normal	2.764	2.556	2.238	3.917	2.214
50	57	4	normal	2.495	1.719	2.472	2.44	2.322
51	51	4	normal	2.226	2.022	2.784	2.583	2.474
52	38	2	normal	2.226	2.022	2.784	2.583	2.78
53	42	3	normal	2.226	2.022	2.784	2.583	2.627
54	35	2	normal	2.226	2.022	2.784	2.583	2.78
55	46	3	normal	2.495	2.289	3.029	2.97	3.014
56	39	2	LSIL	1.15	0.954	0.768	1.595	0.945
57	37	2	LSIL	1.15	0.954	0.768	1.595	0.945
58	48	3	LSIL	1.15	0.384	1.247	0.505	1.287
59	47	3	LSIL	1.15	0.384	1.247	0.505	1.287
60	20	1	LSIL	1.15	0.384	1.247	0.505	1.593
61	42	3	LSIL	1.957	1.755	1.503	2.756	1.953
62	35	2	LSIL	1.544	0.954	1.59	1.599	1.74
63	46	3	LSIL	2.226	2.022	1.748	3.143	1.593

64	49	3	LSIL	1.15	0.954	1.804	1.035	1.826
65	51	4	LSIL	1.15	0.954	0.768	1.595	0.639
66	50	4	LSIL	1.15	0.954	1.804	1.035	1.673
67	61	5	LSIL	1.15	0.954	1.804	1.035	1.52
68	21	1	LSIL	1.15	0.954	1.804	1.035	2.132
69	48	3	LSIL	1.15	0.954	1.804	1.035	1.826
70	34	2	LSIL	1.15	0.954	1.804	1.035	1.979
71	36	2	LSIL	2.135	0.954	2.046	2.025	2.157
72	32	2	LSIL	1.544	0.954	2.108	1.319	2.257
73	54	4	LSIL	1.544	0.954	2.108	1.319	1.951
74	51	4	LSIL	1.544	0.954	2.108	1.319	1.951
75	34	2	LSIL	2.495	2.289	2.511	3.25	2.65
76	57	4	LSIL	2.135	0.954	2.564	1.745	2.368
77	84	5	LSIL	2.135	0.954	2.564	1.745	2.215
78	29	1	LSIL	2.226	2.022	2.784	2.583	2.933
79	23	1	LSIL	2.226	2.022	2.784	2.583	2.933
80	54	4	LSIL	2.495	2.289	3.029	2.97	2.861
81	24	1	LSIL	3.48	2.289	3.271	3.96	3.498
82	43	3	LSIL	3.014	2.022	3.392	3.151	3.183
83	39	2	LSIL	3.211	2.194	3.544	3.293	3.475
84	47	3	LSIL	3.48	2.289	3.789	3.68	3.709
85	51	4	LSIL	3.552	2.728	3.882	3.925	3.804
86	24	1	LSIL	3.552	2.728	3.882	3.925	4.263
87	54	4	HSIL	1.15	0.954	0.768	1.595	0.639
88	52	4	HSIL	1.15	0.954	0.768	1.595	0.639
89	35	2	HSIL	1.15	0.384	1.247	0.505	1.44
90	68	5	HSIL	1.957	1.755	1.503	2.756	1.647
91	66	5	HSIL	2.495	2.289	1.993	3.53	1.674
92	34	2	HSIL	2.495	2.289	1.993	3.53	2.133
93	56	4	HSIL	2.495	2.289	1.993	3.53	1.827
94	26	1	HSIL	3.408	1.624	2.103	3.465	2.194
95	44	3	HSIL	2.764	2.556	2.238	3.917	2.367
96	47	3	HSIL	2.135	0.954	2.564	1.745	2.521
97	51	4	HSIL	2.135	1.126	2.564	1.745	2.368

98	31	2	HSIL	4.412	2.425	2.99	4.768	3.341
99	33	2	HSIL	4.412	2.425	2.99	4.768	3.341
100	38	2	HSIL	4.143	2.728	3.302	4.911	3.493
101	28	1	HSIL	4.412	2.995	3.547	5.298	4.033
102	39	2	HSIL	4.412	2.995	3.547	5.298	3.88
103	68	5	HSIL	4.143	2.158	3.781	3.821	3.529
104	55	4	HSIL	3.48	2.461	3.789	3.68	3.556
105	49	3	HSIL	3.48	2.461	3.789	3.68	3.709
106	51	4	HSIL	4.215	2.425	3.874	4.066	3.93
107	43	3	HSIL	3.677	2.461	3.941	3.822	3.848
108	61	5	HSIL	3.749	2.728	4.034	4.067	3.79
109	38	2	HSIL	3.749	2.728	4.034	4.067	4.249
110	47	3	HSIL	3.946	2.728	4.186	4.209	4.235
111	51	4	HSIL	3.946	2.728	4.186	4.209	4.082
112	32	2	HSIL	4.143	2.728	4.338	4.351	4.527
113	51	4	HSIL	4.143	2.728	4.338	4.351	4.221
114	39	2	HSIL	4.412	2.995	4.583	4.738	4.914
115	54	4	HSIL	4.412	2.995	4.583	4.738	4.608
116	34	2	HSIL	4.412	2.995	4.583	4.738	4.914
117	52	4	HSIL	4.412	2.995	4.583	4.738	4.608
118	33	2	HSIL	4.412	2.995	4.583	4.738	4.914
119	47	3	HSIL	4.412	2.995	4.583	4.738	4.761
120	47	3	HSIL	4.412	2.995	4.583	4.738	4.761
121	47	3	SCC	2.529	1.126	2.868	2.029	2.799
122	82	5	SCC	2.529	1.126	2.868	2.029	2.493
123	34	2	SCC	4.412	2.995	3.547	5.298	3.88
124	60	4	SCC	4.412	2.995	3.547	5.298	3.574
125	60	4	SCC	4.215	2.995	4.431	4.596	4.469
126	51	4	SCC	3.874	2.461	4.093	3.964	3.834
127	58	4	SCC	4.412	2.995	4.583	4.738	4.608
128	62	5	SCC	4.412	2.995	4.583	4.738	4.455
129	45	3	SCC	4.412	2.995	4.583	4.738	4.761
130	55	4	SCC	4.412	2.995	4.583	4.738	4.608

Supplement Table S3. Sensitivity, specificity, PPV and NPV of pathological grades analyzed by models 1 to 5 in the test sample set.

	Tested sample set				
	LSIL group (Normal+LSIL) *vs* HSIL group (HSIL+SCC)				
	**Sensitivity**	**Specificity**	**PPV**	**NPV**	**OR**
Model 1	86	93	73	97	77.52
Model 2	91	90	67	98	87.75
Model 3	91	96	85	98	254.89
Model 4	93	91	69	98	127.40
Model 5	91	96	83	98	221.81

PPV = Positive predictive value, NPV = Negative predictive value, OR = odds ratio

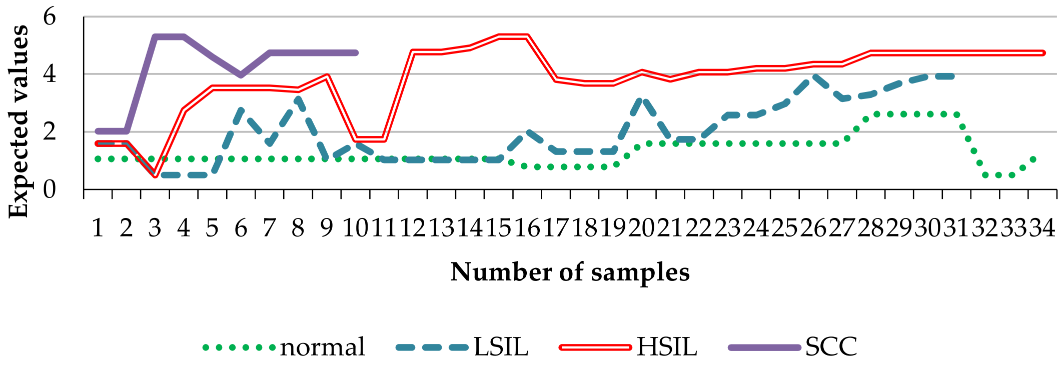

Supplement Figure S1. Means of the expected values from the linear regression models and clinical pathological grades (each sample) of confirmed sample sets.

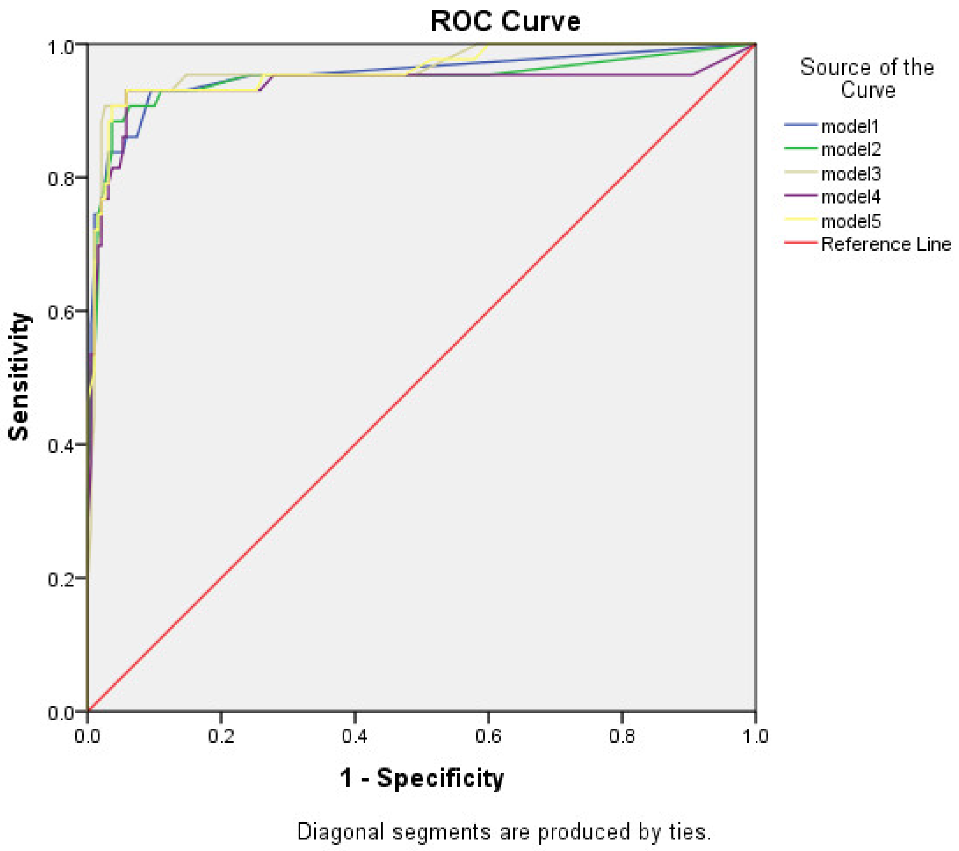

Supplement Figure S2. ROC curves and AUCs when comparing sensitivity and specificity of models 1 to 5 between normal+LSIL and HSIL+SCC in the test sample set. AUCs of models 1 to 5 were 0.953, 0.946, 0.962, 0.937 and 0.960, respectively (*P*=0.000).

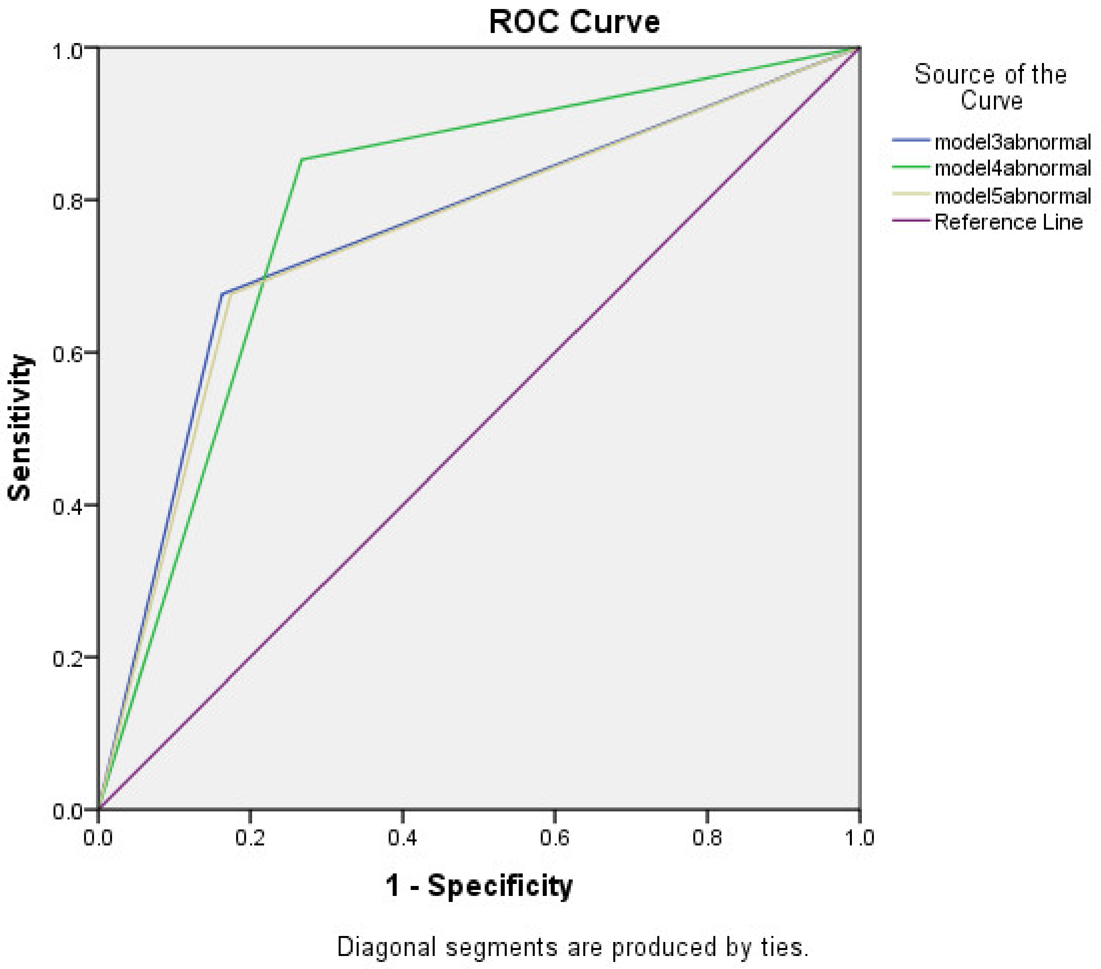

Supplement Figure S3. ROC curves and AUC when comparing sensitivity and specificity of models 3 to 5 between normal and LSIL+HSIL in a confirmed sample set. AUCs of these models were 0.757, 0.793, and 0.751, respectively (*P*=0.000) to predict risk biomolecules.

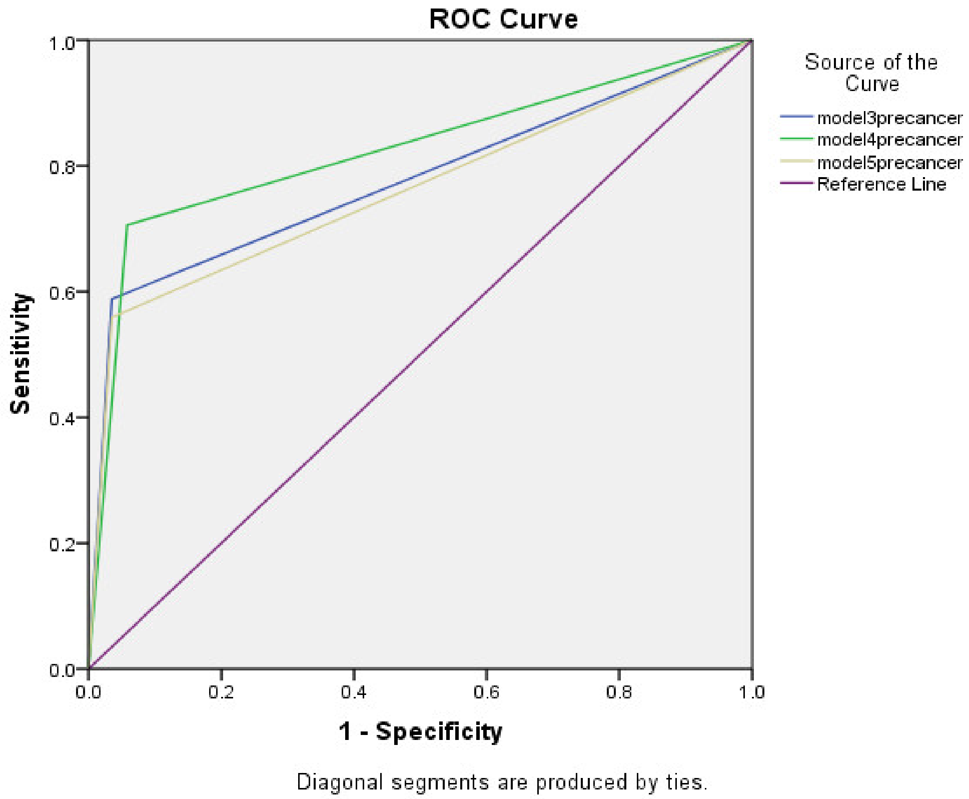

Supplement Figure S4. ROC curves and AUCs when comparing sensitivity and specificity of models 3 to 5 between normal+LSIL and HSIL in a confirmed sample set. AUCs of these model were 0.777, 0.824 and 0.762, respectively (*P* =0.000) to predict precancerous lesions.

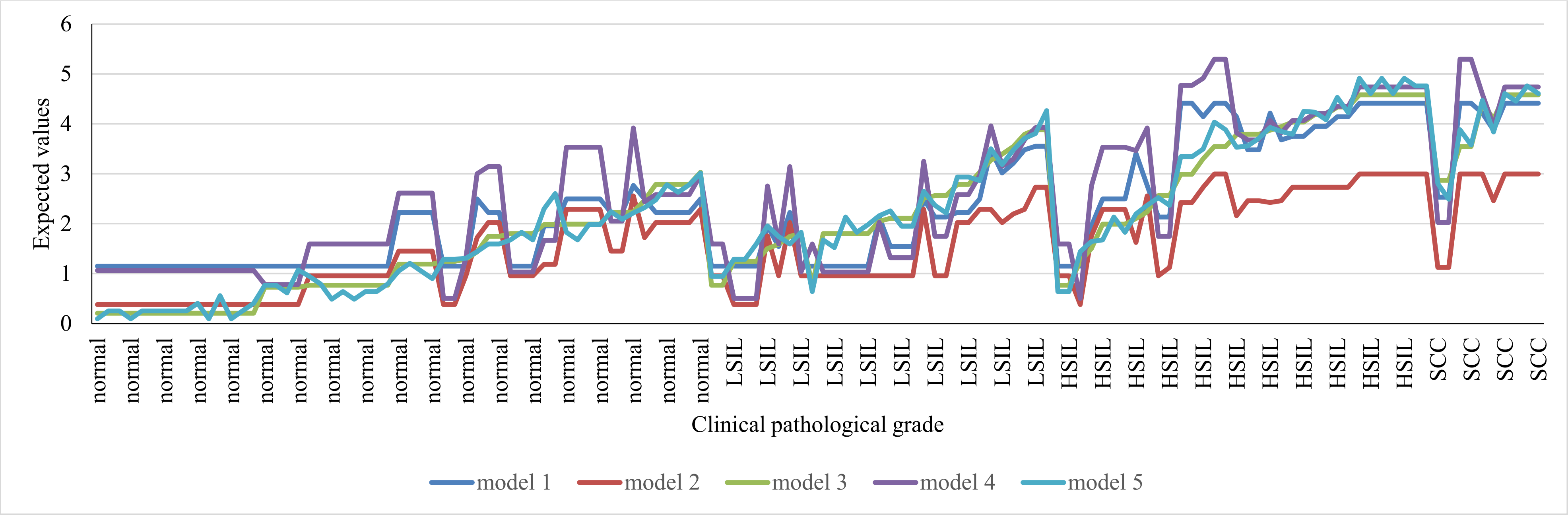

Supplement Figure S5. Means of the expected values from the 5 linear regression models and clinical pathological grades (each sample) of confirmed sample sets.
